# Thermographic Imaging of the Superficial Temperature in Racing Greyhounds before and after the Race

**DOI:** 10.1100/2012/182749

**Published:** 2012-10-14

**Authors:** Mari Vainionpää, Esa-Pekka Tienhaara, Marja Raekallio, Jouni Junnila, Marjatta Snellman, Outi Vainio

**Affiliations:** ^1^Department of Equine and Small Animal Medicine, Pharmacology and Toxicology, Faculty of Veterinary Medicine, University of Helsinki, Koetilantie 7, 00014 Helsinki, Finland; ^2^Kosonkuja 3, 04370 Rusutjärvi, Finland; ^3^4Pharma Ltd, 02130 Espoo, Finland

## Abstract

A total of 47 racing greyhounds were enrolled in this study on two race days (in July and September, resp.) at a racetrack. Twelve of the dogs participated in the study on both days. Thermographic images were taken before and after each race. From the images, superficial temperature points of selected sites (*tendo calcaneus, musculus gastrocnemius, musculus gracilis,* and *musculus biceps femoris portio caudalis*) were taken and used to investigate the differences in superficial temperatures before and after the race. The thermographic images were compared between the right and left legs of a dog, between the raced distances, and between the two race days. The theoretical heat capacity of a racing greyhound was calculated. With regard to all distances raced, the superficial temperatures measured from the *musculus gastrocnemius* were significantly higher after the race than at baseline. No significant differences were found between the left and right legs of a dog after completing any of the distances. Significant difference was found between the two race days. The heat loss mechanisms of racing greyhounds during the race through forced conduction, radiation, evaporation, and panting can be considered adequate when observing the calculated heat capacity of the dogs.

## 1. Introduction

Racing greyhounds have been bred for the purpose of competitive racing for centuries [1]. This selective breeding has influenced the biochemical properties of racing greyhounds as well as their musculature and appearance [1, 2]. Due to their unique ability to run fast, the dogs also have different energy requirements especially for race performance [3]. The efficiency of the physical exertion with the muscles is a measure of the body's effectiveness in converting nutrients' energy into external muscle work [1, 2]. In humans, the efficiency of muscle work has been studied and estimated to be 24.4%–34.0% [4, 5]. Similar data is not readily available for racing greyhounds.

Running speed has yielded a wind effect during the race and influences the convection of body heat. Furthermore, the vasodilation of the peripheral vascular system is an important way of releasing heat from the body [6]. Other ways for the heat of the body to escape are radiation, evaporation, conduction, and panting [6–8]. 

Thermographic imaging (also known as thermography, infrared thermography, and infrared imaging) can be used to measure superficial temperature changes in the body [9]. Compared to other commonly used diagnostic methods, thermographic imaging is a noninvasive and safe method of detecting and visualizing changes in superficial temperature in animals [10–14]. A change in superficial temperature due to changes in peripheral blood flow can be measured feasibly with thermography [9]. The method has been used to study dogs [15–17], horses [10, 11, 13], farm animals [12], and humans [18–20]. Thermography can be used to detect, for example, muscle injuries in dogs [21] since such injuries cause a change in peripheral blood flow.

Studies on humans suggest that in healthy individuals, the difference in the symmetry of superficial temperatures between the left and right side is approximately 0.4°C ± 0.3°C [22]. Differences greater than those found in healthy humans could be considered abnormal. No similar studies have been done on animals, but we hypothesized that asymmetry in the superficial temperatures detected by means of thermography may be indicative of adverse events during the race, such as asymmetric strain on the legs or trauma in racing greyhounds. Our secondary hypothesis was that thermography can also be used to detect exercise-induced vasodilatation and increased heat production in racing greyhounds. To our knowledge, thermographic imaging has not been applied before to study the heat balance of healthy racing greyhounds.

The aim of this study was to investigate the differences in superficial temperature on selected points of the muscles and tendons in racing greyhound before and after the race by means of thermographic imaging. The secondary aim was to compare the thermographic images between the right and left legs of individual dogs, as well as between the raced distances and between the two race days. Additional aims were to calculate the theoretical heat capacity of a racing greyhound and measure the relationships between the two race days as well as between the left and right legs with two predetermined factors (kinetic energy and rank) by using the data obtained from the thermographic images and the race records. Our hypotheses were that the superficial temperature measured by thermographic imaging would change due to exercise and that change between the right and left legs would be minor. The superficial temperature difference before and after the race is an important indicator of the well-being of the racing greyhound.

## 2. Materials and Methods

### 2.1. The Dogs

Forty-eight racing greyhounds were enrolled in this study at two races, in July and in September, respectively. The owners of the dogs were asked for consent to participate before enrolment. The dogs were between 15 months and 8 years of age and weighted, on average, 32.4 kg, range 24.7–39.5 kg. All the dogs were preliminarily considered to be healthy since they were participating in a race. The dogs also had to go through a health check performed by a veterinarian before being cleared to race. The dogs and runs with notably incomplete data from the race records or thermographic images were excluded (*n* = 1 and *n* = 8, resp.) from the study, thus leaving a total of 47 dogs and 59 races used in the statistical calculations. Twelve of the 47 dogs participated in the study on both days.

### 2.2. Thermographic Imaging

**Figure 1:**
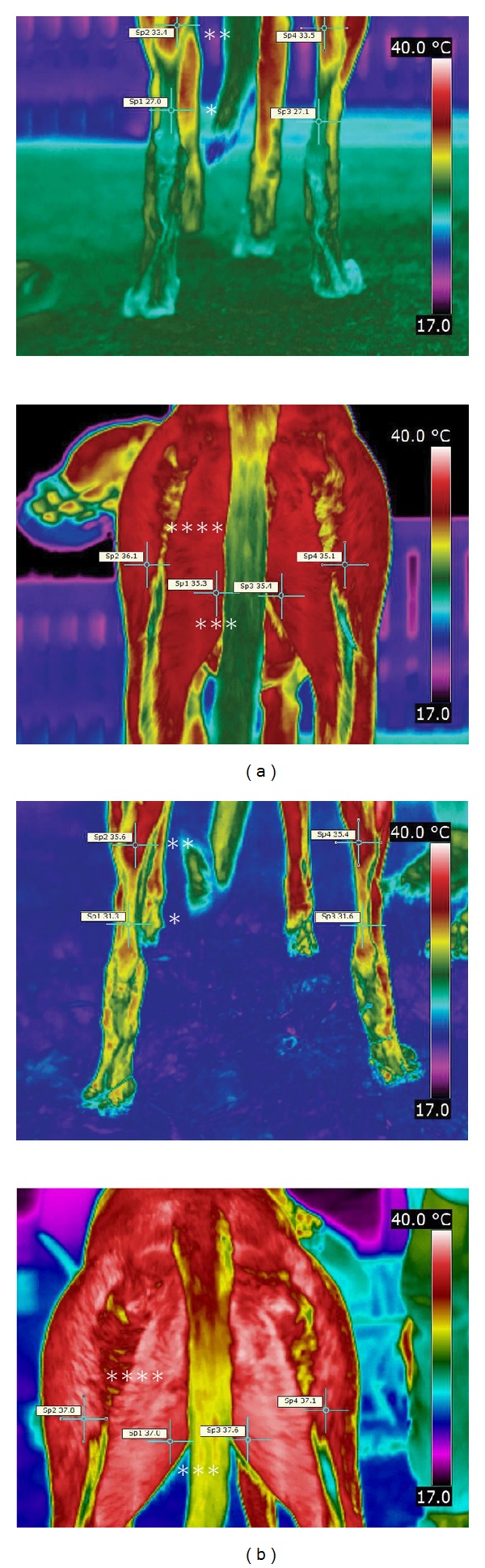
Thermographic images before (a) and after (b) the race, showing the four measurement points of the *tendo calcaneus* (*), *musculus gastrocnemius* (**), *musculus gracilis* (***), and *musculus biceps femoris portio caudalis* (****).

All the thermographic images were taken with a thermal camera with a resolution of 320 by 240 pixels (T425, FLIR Systems Inc., Danderyd, Sweden). The high-rainbow Color Palette was chosen for taking and displaying the images. The emissivity of one (*e* = 1) was set for the imaging. The thermographic images from the caudal part of the dog, one distally and one proximally (Figure 1), were taken before the veterinary checkup outside with no shelter from the wind or sun due to the lack of a covered space in the area and again within 5 minutes after the race in a large tent set up beside the racetrack near the gate where the racing dogs exited after the race.

### 2.3. Racetrack and Racing Distances

The thermographic imaging was carried out at a racetrack in Turku, Finland. The track is 475 metres long and has a diameter of the curve of 104 metres.

The dogs in this study were racing in four different distances: 325, 495, 560, and 785 metres. For the 325-metre sprint, there were a total of 27 runs, and for the 495, 560, and 785-metre sprints, 17, 9, and 6 runs, respectively. 

### 2.4. Data Analysis

The freeware FLIR QuickReport 2.1. [23] was applied to interpret the thermographic images. One veterinarian blinded to the dog in question analysed all the images. From the images, four superficial temperature measurement points were selected: *tendo calcaneus, musculus gastrocnemius, musculus gracilis,* and *musculus biceps femoris portio caudalis*. The thermal symmetry was studied from the thermographic images by means of calculating the superficial temperatures of the *tendo calcaneus* and the *musculus gastrocnemius* as well as the *musculus gracilis* and *musculus biceps femoris portio caudalis* before and after the race (Figure 1). The measurement sites were chosen because the areas of interest are affected by physical exercise and they are easily accessed and seen with thermal imaging.

The ambient temperature was measured with a reference thermometer (Fluke T524, Fluke corporation, USA) when available. In July, this measurement device was not available to us, and the ambient temperature was measured from the thermal images by determining the coldest spots in the background temperatures in the four images taken from a specific dog, after which the warmest of these cold spots was selected for calculations. The average ambient temperature on the first race day in July was 18.3°C (13.1–23.3°C) and on the second race day in September 15.7°C (14.5–16.1°C).

The velocity of the sprint was calculated using the formula *v* = *s*/*t*, where *s* = distance (metres), *t* = time (seconds). Kinetic energy was calculated using the formula (1/2)  *m*∗*v* ^2^, where *m* = mass (kilograms), *v* = velocity (metres/second). Heat capacity was calculated using the formula C*m, where *C* = 4,186 J/(kg∗K) = constant (heat capacity of water), *m* = dog weight (kilograms). The efficiency of muscle work was estimated to be 30% for the calculations [4, 5]. 

### 2.5. Statistical Analysis

Descriptive statistics were constructed for superficial temperatures based on the thermographic images by time point (before race versus after race), leg, day, and distance for each site (*tendo calcaneus, musculus gastrocnemius, musculus gracilis,* and *musculus biceps femoris portio caudalis*). The differences between the time points, legs, and days in the superficial temperatures based on the thermographic images were evaluated with analysis of variance for repeated measures (RM ANOVA) separately for each measurement point. As two of the different distances had data from only one day and two of the distances from two days, the effects of day and distance were confounded. Therefore, the modelling had to be performed separately for each distance. With the two shortest distances (325 and 495 metres), the statistical model included time point, leg, day, and an interaction term of time point and leg as fixed effects and dog as a random effect. With the two longer distances (560 and 785 metres), the model was the same, excluding the effect of day. 

The relationship between the difference between the prerace and postrace superficial temperatures and the predetermined explaining factors was assessed with ANOVA models separately for each measurement point. Based on the primary models, the difference between the legs was negligible with all locations and distances. Therefore, this analysis was only conducted for the difference between time points. The response variable was the mean change in the superficial temperature (before and after) of the two thermographically imaged legs of the dog. The fitted statistical model included (for the two shortest distances) day, rank, and kinetic energy as fixed effects. For the 560 metre distance, the model was the same, excluding the effect of day. For the longest distance, there was not enough data available on the different starts for the evaluation of the effect of rank. Therefore, the model for the longest distance included only the effect of kinetic energy.

All statistical analyses were carried out using the SAS System for Windows, version 9.3 (SAS Institute Inc., Cary, NC, USA).

This study was approved by the Ethical Committee of the Viikki Campus at the University of Helsinki.

## 3. Results

After all of the distances raced, the superficial temperatures were significantly higher than those at baseline in *musculus gastrocnemius* (Table 1). The superficial temperature differences measured from other points before and after the race varied depending on the measurement point (Table 1). The estimates of the superficial temperature difference varied from 0.6 to 2.1 degrees depending on the distance raced. The superficial temperature differences between left and right leg varied between 0.0 and 4.0 degrees. No significant differences were found between the two thermographically imaged legs of the dog after any of the distances and measurement points. No clinical signs of obvious injuries were detected by the veterinarian responsible for the racing dogs during or after the race.

After the 325- and 495-metre sprints, a significant difference was found between the two race days with all of the measurement points, excluding the 325-metre sprint from *musculus biceps femoris portio caudalis*. No significant differences were detected between rank and kinetic energy when compared to the pre- and postrace superficial temperatures for any of the distances or any of the measurement points. The calculated heat capacity values were similar for all of the distances run and both race days. Data on the dogs, race, velocity, kinetic energy, and heat loss calculations are shown in Table 2.

## 4. Discussion

After the race, the superficial temperatures of the greyhounds, as evaluated by means of thermographic images, were significantly higher than those before the race for all the distances raced on the *musculus gastrocnemius,* and after some of the distances also on the other measurement points. The superficial temperature change was predictable since the muscle work during the race generated a considerable amount of heat as seen in the heat capacity calculations. Furthermore, the subsequent vasodilation of the superficial vasculature and the distribution of the blood have an effect. However, the circumstantial factors on the day of the race also seemed to be important in explaining the change in the superficial temperatures. On the race day in July, the ambient temperature was higher than that on race day in September. 

The amount of heat generated during the run (heat capacity) was calculated based on known constants (*C*), the body weight of the dog, and the calculated kinetic energy. Although this was a theoretical count, it indicated that heat production during the race was considerable and would have led to hyperthermia without proper cooling systems in the dog. It has been shown in previous studies that dogs lose heat *via* conductance through the tissue [6] and respiratory evaporation [8]. Our findings indicate that these methods of heat loss are most likely not sufficient but, additionally, that heat is lost *via* convection and the wind effect generated by the velocity of the dog, a forced convection. The small amount of body fat in racing greyhounds [2] and the lack of or very thin hair coat over the whole abdomen and hind legs contribute to the heat loss, since it is known that the thickness of the hair coat has an effect on the insulation capacity [7]. Since the dogs did not show any signs of hyperthermia after the race, the heat loss during the race was probably adequate in the ambient temperatures of the present study. The dogs expressed no signs of greater discomfort on the warmer race day in July when compared to the race day in September. This also suggests that the cooling mechanisms of racing greyhounds are functional regardless of the ambient temperature. The ambient temperatures on the two race days were low compared to those in many other countries. Despite the warmer ambient temperatures, racing greyhounds are known to complete the race without severe hyperthermia.

No significant differences were detected in the means of the superficial temperatures between the right and left legs of the dogs, despite of the effects of the counterclockwise race direction, suggesting that in general the dogs had been running in a balanced manner. Unilateral injuries can be seen in superficial temperature differences between the left and right sides in thermographic images [11, 22]. However, superficial temperature differences up to 4.0 degrees were detected between right and left legs after the race without any obvious sign of injury. Unbalanced use of the muscles can also be seen as superficial temperature differences between the sides. The characteristics of the racetrack have an effect on injuries occurring during the races, and the speed of the dog and the raced distance are also known to influence the injury rate [24]. However, we could not evaluate such associations due to the low incidence of injuries detected by means of thermographic imaging in our material.

As the current data was not balanced (data on some distances obtained from two days and on others from just one day), a full evaluation of the differences between the distances could not be conducted. Another limitation of the present study was the rather low number of dogs, decreasing the statistical power, especially when analysing the data for the two longest distances. The low number of dogs also affected the evaluation of the effects of the explanatory variables, rank and kinetic energy. The fact that this study was conducted during an actual race with racing dogs may have caused some minor bias due to the uncontrolled environment. For example, the amount of warm-up exercise before the first thermographic imaging was not known due to the individual and private training protocols of the dogs. Even though the amount of warm-up exercise before the first thermographic imaging was not known, the dogs were queuing for at least 10 minutes before the health check, during which time the thermographic images were taken. This waiting period before the imaging most likely levels out the differences between the dogs.

In conclusion, thermographic imaging appears to be a useful tool for measuring superficial temperatures in racing greyhounds in the race setting. Increases in the superficial temperatures induced by racing could be detected by thermographic imaging. This result was predictable since the muscle work during the race generates heat. No differences were seen in the superficial temperature between the left and right legs after the race, suggesting balanced muscle work and an appropriate racetrack for racing greyhounds. The calculated heat capacity was similar for all distances raced and for both race days. In spite of the amount of heat generated during the race, the dogs did not develop signs of severe hyperthermia, which suggests that the heat loss during the race through forced convection, conduction, radiation, evaporation, and panting was adequate. Our result suggests that thermographic imaging can be used to monitor heat-balance-related well-being of the racing greyhound. However, the method of thermographic imaging needs to be studied further for more advanced applications for racing greyhounds. 

## Figures and Tables

**Table 1:** Averages ± SD as well as minimum (min) and maximum (max) superficial temperatures obtained from the thermographic images. Measurement points (MPs) for the left and right leg: (1) *musculus* *gastrocnemius,* (2) *tendo* *calcaneus,* (3) *musculus* *biceps* *femoris* *portio* *caudalis*, and (4) *musculus* *gracilis*. *P* values are for differences before and after the race for each measurement point and distance estimated from the primary statistical models.

			Thermographic images (*t*°C)				
			Before race		After race		
Day	Meters	MP	Left leg	Right leg	Left leg	Right leg	*P* value
			Mean ± SD (min, max)	Mean ± SD (min, max)	Mean ± SD (min, max)	Mean ± SD (min, max)	
July	325	1	33.8 ± 1.2 (32.2, 35.6)	33.8 ± 1.3 (31.9, 35.5)	34.5 ± 1.7 (31.6, 37.4)	34.5 ± 1.7 (31.0, 37.7)	
Sep.	325	1	31.6 ± 1.9 (26.2, 33.8)	31.5 ± 2.0 (25.7, 33.4)	32.0 ± 1.4 (29.3, 34.8)	33.1 ± 1.1 (31.6, 35.0)	0.002
July	325	2	30.6 ± 2.1 (26.6, 33.1)	30.7 ± 2.2 (26.9, 33.9)	29.5 ± 2.3 (26.8, 33.2)	29.6 ± 2.3 (26.3, 34.4)	
Sep.	325	2	24.7 ± 4.3 (18.1, 32.6)	24.1 ± 4.4 (14.8, 31.0)	26.3 ± 3.0 (18.4, 29.8)	27.1 ± 3.3 (18.6, 30.4)	0.285
July	325	3	33.2 ± 2.5 (28.4, 36.2)	33.3 ± 2.4 (28.9, 36.6)	32.8 ± 3.7 (26.4, 37.9)	33.3 ± 3.8 (26.2, 38.7)	
Sep.	325	3	29.9 ± 3.7 (23.4, 35.1)	29.8 ± 3.4 (22.9, 35.7)	31.6 ± 3.3 (25.9, 36.8)	31.5 ± 3.1 (25.3, 36.0)	0.043
July	325	4	35.6 ± 0.8 (34.0, 36.8)	35.5 ± 1.0 (32.7, 36.9)	36.6 ± 1.1 (35.1, 38.3)	36.6 ± 0.9 (35.5, 38.4)	
Sep.	325	4	32.8 ± 3.1 (25.2, 35.9)	32.9 ± 2.6 (27.7, 35.9)	34.7 ± 1.4 (31.2, 36.4)	34.3 ± 1.1 (31.6, 35.9)	<0.001

July	495	1	34.6 ± 1.1 (32.5, 36.2)	34.4 ± 1.0 (32.7, 35.8)	34.9 ± 1.0 (33.4, 36.5)	35.2 ± 0.9 (33.6, 36.4)	
Sep.	495	1	31.1 ± 0.8 (29.9, 31.5)	31.5 ± 0.6 (31.0, 32.4)	31.2 ± 1.3 (29.4, 32.6)	32.7 ± 0.9 (31.6, 33.8)	0.003
July	495	2	30.6 ± 2.1 (27.0, 34.0)	31.2 ± 2.5 (27.1, 35.1)	28.9 ± 1.7 (25.4, 31.7)	29.8 ± 1.8 (26.1, 32.5)	
Sep.	495	2	24.5 ± 3.6 (20.7, 29.0)	24.6 ± 3.3 (21.5, 27.9)	23.2 ± 4.1 (18.5, 28.6)	24.8 ± 4.0 (21.3, 30.6)	0.003
July	495	3	34.9 ± 1.4 (31.1, 36.6)	35.0 ± 1.2 (32.0, 36.6)	34.7 ± 2.1 (29.7, 37.8)	34.9 ± 1.7 (31.7, 38.2)	
Sep.	495	3	30.3 ± 2.7 (27.7, 34.0)	28.4 ± 2.5 (26.4, 31.8)	31.5 ± 2.3 (29.8, 34.9)	29.5 ± 3.7 (25.6, 34.5)	0.629
July	495	4	35.7 ± 0.4 (34.8, 36.4)	35.8 ± 0.3 (35.3, 36.2)	36.7 ± 0.8 (35.7, 38.3)	37.0 ± 0.9 (35.7, 38.7)	
Sep.	495	4	34.1 ± 0.7 (32.6, 34.2)	33.9 ± 0.6 (33.2, 34.4)	34.9 ± 2.2 (29.9, 34.4)	34.8 ± 0.4 (33.6, 34.5)	<0.001

July	560	1	33.5 ± 1.2 (31.8, 34.9)	33.7 ± 1.4 (31.4, 35.8)	34.3 ± 1.5 (32.1, 36.4)	34.7 ± 1.3 (32.5, 36.5)	0.005
July	560	2	28.3 ± 1.1 (27.2, 29.8)	28.4 ± 1.0 (27.4, 30.3)	29.4 ± 2.4 (25.6, 32.6)	28.1 ± 4.7 (17.8, 33.4)	0.683
July	560	3	34.7 ± 1.5 (31.1, 35.9)	34.7 ± 1.6 (30.9, 36.2)	34.3 ± 2.9 (30.4, 37.4)	34.3 ± 2.1 (30.5, 36.6)	0.447
July	560	4	35.5 ± 1.3 (33.1, 37.0)	35.3 ± 1.2 (32.6, 37.1)	35.7 ± 1.3 (33.0, 36.8)	36.1 ± 1.4 (33.6, 37.7)	0.115

Sep.	785	1	30.6 ± 1.0 (29.1, 32.2)	30.4 ± 1.0 (29.1, 31.6)	32.6 ± 0.9 (31.4, 33.8)	32.5 ± 0.9 (31.5, 33.4)	<0.001
Sep.	785	2	21.8 ± 3.4 (18.3, 28.3)	22.8 ± 3.2 (18.1, 28.2)	23.9 ± 2.6 (20.5, 28.4)	24.3 ± 3.0 (20.7, 29.4)	0.153
Sep.	785	3	31.9 ± 2.0 (28.9, 34.5)	32.0 ± 1.6 (29.4, 33.6)	33.9 ± 2.2 (31.9, 37.6)	33.1 ± 3.2 (27.9, 37.4)	0.053
Sep.	785	4	33.4 ± 1.4 (31.6, 35.2)	33.4 ± 1.2 (32.1, 35.4)	35.4 ± 1.1 (34.3, 37.1)	35.6 ± 1.2 (34.0, 36.9)	0.005

**Table 2:** Data on the races as well as energy and heat capacity calculations for each dog, distance and race day.

Day	Running distance (m)	Number of dogs	Body mass (kg)	Running time (s)	Velocity (m/s)	Kinetic energy (kJ)	Heat capacity (kJ)
			Ave. ± SD (min, max)	Ave. ± SD (min, max)	Ave. ± SD (min, max)	Ave. ± SD (min, max)	Ave. ± SD (min, max)
July	325	14	33.7 ± 3.3 (29.2, 38.6)	19.6 ± 0.3 (19.3, 20.4)	16.6 ± 0.2 (15.9, 16.7)	4.6 ± 0.5 (4.0, 5.5)	141.4 ± 14.0 (122.4, 161.7)
Sep	325	13	32.9 ± 3.6 (26.9, 39.5)	19.2 ± 0.3 (18.9, 19.7)	16.9 ± 0.2 (16.5, 17.2)	4.7 ± 0.5 (3.8, 5.7)	137.9 ± 15.0 (112.3, 165.5)

July	495	13	31.0 ± 4.1 (25.3, 38.4)	29.6 ± 0.3 (29.3, 30.2)	16.7 ± 0.2 (16.4, 16.9)	4.3 ± 0.6 (3.4, 5.4)	129.8 ± 17.3 (106.0, 160.9)
Sep	495	4	31.5 ± 5.0 (26.1, 35.9)	29.6 ± 0.3 (29.2, 29.9)	16.7 ± 0.2 (16.6, 16.9)	4.4 ± 0.7 (3.7, 5.0)	131.9 ± 20.8 (109.4, 150.4)

July	560	9	32.1 ± 3.9 (25.1, 36.6)	33.4 ± 0.6 (32.5, 33.8)	16.8 ± 0.3 (16.6, 17.3)	4.5 ± 0.6 (3.6, 5.5)	134.4 ± 16.5 (105.2, 153.4)

Sep	785	6	31.8 ± 5.8 (24.7, 38.4)	48.6 ± 0.3 (48.4, 49.1)	16.2 ± 0.1 (16.0, 16.3)	4.1 ± 0.7 (3.2, 4.9)	133.3 ± 22.0 (103.5, 160.9)

## References

[1] Dobson GP, Parkhouse WS, Weber JM (1988). Metabolic changes in skeletal muscle and blood of greyhounds during 800-m track sprint. *American Journal of Physiology*.

[2] Zaldívar-López S, Lopez LM, Iazbik MC, Westendorf-Stingle N, Hensley S, Couto CG (2011). Clinical pathology of Greyhounds and other sighthounds. *Veterinary Clinical Pathology*.

[3] Hill RC, Bloomberg MS, Legrand-Defretin V (2000). Maintenance energy requirements and the effect of diet on performance of racing Greyhounds. *American Journal of Veterinary Research*.

[4] Gaesser GA, Brooks GA (1975). Muscular efficiency during steady rate exercise: effects of speed and work rate. *Journal of Applied Physiology*.

[5] Mogensen M, Bagger M, Pedersen PK, Fernström M, Sahlin K (2006). Cycling efficiency in humans is related to low UCP3 content and to type I fibres but not to mitochondrial efficiency. *Journal of Physiology*.

[6] Hammel HT, Wyndham CH, Hardy JD (1958). Heat production and heat loss in the dog at 8–36°C environmental temperature. *American Journal of Physiology*.

[7] Hammel HT (1955). Thermal properties of fur. *The American Journal of Physiology*.

[8] Taylor CR, Schmidt-Nielsen K, Dmi’el R, Fedak M (1971). Effect of hyperthermia on heat balance during running in the African hunting dog. *The American Journal of Physiology*.

[9] Stewart M, Webster JR, Verkerk GA, Schaefer AL, Colyn JJ, Stafford KJ (2007). Non-invasive measurement of stress in dairy cows using infrared thermography. *Physiology and Behavior*.

[10] Graf von Schweinitz D (1999). Thermographic diagnostics in equine back pain. *The Veterinary Clinics of North America Equine Practice*.

[11] Turner TA (2001). Diagnostic thermography. *The Veterinary Cof North America Equine Practice*.

[12] Kízková I, Kunc P (2007). Applications of infrared thermography in animal production. *Journal of Faculty of Agriculture*.

[13] Levet T, Martens A, Devisscher L, Duchateau L, Bogaert L, Vlaminck L (2009). Distal limb cast sores in horses: risk factors and early detection using thermography. *Equine Veterinary Journal*.

[14] Vainionpää MH, Salla K, Restitutti F Thermographic imaging of superficial temperature in dogs sedated with medetomidine and butorphanol with and without MK-467 (L-659'066).

[15] Loughin CA, Marino DJ (2007). Evaluation of thermographic imaging of the limbs of healthy dogs. *American Journal of Veterinary Research*.

[16] Infernuso T, Loughin CA, Marino DJ, Umbaugh SE, Solt PS (2010). Thermal imaging of normal and cranial cruciate ligament-deficient stifles in dogs. *Veterinary Surgery*.

[17] Marino DJ, Loughin CA (2010). Diagnostic imaging of the canine stifle: a review. *Veterinary Surgery*.

[18] Varjú G, Pieper CF, Renner JB, Kraus VB (2004). Assessment of hand osteoarthritis: correlation between thermographic and radiographic methods. *Rheumatology*.

[19] Ng EYK, Kee EC (2008). Advanced integrated technique in breast cancer thermography. *Journal of Medical Engineering and Technology*.

[20] Tan JH, Ng EYK, Rajendra Acharya U, Chee C (2009). Infrared thermography on ocular surface temperature: a review. *Infrared Physics and Technology*.

[21] Steiss JE (2002). Muscle disorders and rehabilitation in canine athletes. *Veterinary Clinics of North America*.

[22] Vardasca R, Ring F, Plassmann P, Jones C (2012). Termal symmetry of the upper and lower extremities in healthy subjects. *Thermology International*.

[23] http://www.flir.com/cs/emea/en/view/?id=42406.

[24] Sicard GK, Short K, Manley PA (1999). A survey of injuries at five greyhound racing tracks. *Journal of Small Animal Practice*.

